# Immunogenicity of COVID-19 vaccines in patients with diabetes mellitus: A systematic review

**DOI:** 10.3389/fimmu.2022.940357

**Published:** 2022-08-29

**Authors:** Amir Bahador Boroumand, Mahtab Forouhi, Farzaneh Karimi, Arman Soltani Moghadam, Leila Ghanbari Naeini, Pajman Kokabian, Delaram Naderi

**Affiliations:** ^1^ Department of Emergency Medicine, School of Medicine, Isfahan University of Medical Sciences, Isfahan, Iran; ^2^ Department of Clinical Pharmacy, School of Pharmacy, Shahid Beheshti University of Medical Sciences, Tehran, Iran; ^3^ Behbahan Faculty of Medical Sciences, Behbahan, Iran; ^4^ Islamic Azad University, Tehran, Iran; ^5^ Gulf Medical University, Ajman, United Arab Emirates; ^6^ Shahid Beheshti University of Medical Sciences, Tehran, Iran; ^7^ Student Research Committee, Allied Medical Sciences, Iran University of Medical Sciences, Tehran, Iran

**Keywords:** COVID-19, SARS-CoV-2, diabetes mellitus, vaccination, immunogenicity

## Abstract

**Purpose:**

To evaluate the immunogenicity of COVID-19 vaccines in patients with diabetes mellitus (DM) through a systematic approach.

**Method:**

A comprehensive search was conducted in PubMed, Scopus, and Web of Science with no time restrictions. The search was based on the three main concepts: Covid-19, Vaccine immunogenicity and Diabetes Mellitus.

**Results:**

After excluding irrelevant studies, 16 studies remained for the quantitative assay. Among the sixteen studies, eleven had controls. Type of diabetes was specifically mentioned in six studies (T2DM; n=4, T1DM and T2DM; n=2). Twelve of the included studies were conducted on the immunogenicity of vaccines that included mRNA vaccines (i.e. BNT162b2 and mRNA-1273) in DM, five studies included vector-based vaccines (i.e. Ad5-nCoV and ChAdOx1-S), and five studies assessed the immunogenicity of vaccines in DM, including inactivated vaccines (i.e. BBV-152, CoronaVac, Sinopharm or SinoVac). Most of the current studies indicate lower antibody response in patients with DM compared to individuals without DM, after the second dose of vaccine and irrespective of vaccine type. Several studies have shown that higher age and higher BMI are associated with lower antibody response, while optimum glycemic control and higher GFR are associated with higher antibody response among patients with DM.

**Conclusion:**

Immunogenicity of the vaccines has mostly been reported to be lower among patients with DM compared to healthy controls. There are also few studies assessing variables that significantly affect this association, including age, type of diabetes, BMI, glycemic control and eGFR. Investigating these associations could help us provide the most advantageous condition for patients with DM before, during and after vaccination for optimum antibody response. Many unresolved issues concerning potential factors affecting vaccine immunogenicity, including type of vaccine, numbers of administered doses, re-vaccination intervals and hyperglycemia in patients with DM need to be addressed through future research.

## 1 Introduction

Diabetes mellitus (DM) is a major concern in healthcare worldwide, with high morbidity and mortality. Underlying DM is a significant risk factor for higher susceptibility to coronavirus disease 2019 (COVID-19) with a more severe condition, worse outcomes, and higher mortality (1, 2). There are several possible pathophysiologic explanations for the link between diabetes and COVID-19, including inflammation, activation of renin–angiotensin–aldosterone system (RAAS), and changes in glucose hemostasis and immune response (2–9). Due to the severity of the infection in patients with DM, prevention remains the mainstay.

Timely and appropriate vaccination is a crucial step in primary prevention of risks associated with COVID-19 in patients with DM. Since the initiation of the COVID-19 pandemic, there have been global efforts to develop SARS-CoV-2 vaccines. Different types of vaccines have been introduced so far; the mRNA vaccines [i.e. mRNA-1273 (10) and BNT162b2 (11)], vector based vaccines [i.e. AZD1222 (ChAdOx1) (12), Sputnik V vaccine (GamCOVID-Vac) (13), JNJ-78436735 or Ad26.COV2.S (14)] and inactivated virus (15) [CoronaVac, COVAXIN (BBV152)]. The overall efficacy and safety of COVID-19 vaccines in phase III trials were promising (10), sparking global hope toward ending the current outbreak. Although the efficacy of COVID-19 vaccines has been assessed among the population, including patients with DM, but subgroup analysis has been conducted mostly among high risk patients as a whole and not patients with DM in particular (16–18). The application of COVID-19 vaccines in patients with diabetes remains an ongoing debate.

Pneumococcal pneumonia, influenza, and hepatitis B vaccination are recommended for patients with DM due to sufficient antibody response, decreased hospitalization, complications, and death (19–23). Regarding antibody response against SARS-CoV-2 in COVID-19 patients with DM (24–26), and COVID-19 vaccination, several previous studies have evaluated the immunogenicity of COVID-19 vaccines in patients with DM, but the results have been rather conflicting (27–35).

In this systematic review, we aimed to assess the immunogenicity of COVID-19 vaccines and its’ associated factors in patients with DM. This review could help provide a better insight into decision-making in this group of high-risk patients and unravel gaps in the literature and unresolved issues regarding COVID-19 vaccination in patients with DM for future research.

## 2 Material and methods

### 2.1 Protocol

The study protocol was developed based on the PRISMA guideline. Moreover, the inclusion and exclusion criteria of the participants, studies, intervention and outcome (PICO questions) were determined (26).

### 2.2 Search strategy

PubMed, Scopus, and Web of Science were searched for relevant articles published up to April 27, 2022, matching the PICO question using the following keywords: [(COVID-19) OR (SARS-CoV-2) OR (novel coronavirus) OR (2019-nCoV)] AND [(vaccine) OR (vaccination) OR (vaccinated) OR (immunization)] AND [(Diabetes Mellitus) OR ((Diabetes) AND (Mellitus)) OR (Diabetic)]. English original articles that assessed the immunogenicity of COVID-19 vaccines in patients with DM were included. No limitation was determined for the date or status (i.e., online first or published) of the publication. Moreover, the reference lists were screened for remaining relevant studies and included in case of eligibility. Two reviewers independently performed the literature search, and any disagreement was resolved according to the consensus.

### 2.3 Eligibility criteria

The studies that assessed vaccination in patients with DM were eligible for inclusion. The inclusion criteria were as follows: 1) Population: articles on human subjects; with participants with DM; whose diagnosis of DM was established by an endocrinologist based on ADA criteria (36), 2) Intervention: COVID-19 vaccination, 3) Study design: all retrospective and prospective studies as well as clinical trials, 4) Outcomes: the main outcome of this study was immunogenicity of vaccination in patients with DM. This study defined immunogenicity as the percentage of vaccinated patients who showed positive seroconversion (i.e., COVID-19 antibody levels above the cutoff point). The exclusion criteria were as follows: 1) books, reviews and personal opinions, 2) articles not written in English. Type of DM, publication time and participants’ age were not limited.

### 2.4 Data collection

Eligible studies were evaluated by two experts independently. The following data were extracted from each included publication: title, author, time of publication, country of origin, study design, number of participants at baseline and follow-up, if applicable, clinical subgroups, mean age and male to female ratio of the participants, type of DM, complications of DM, type of COVID-19 vaccination, the immunogenicity of the administered vaccine. Any conflicts in data extraction were discussed or consulted by a third expert and resolved.

### 2.5 Quality assessment

For the quality assessment, we used the National Institutes of Health (NIH) quality assessment tool to evaluate the included studies. The scores of 0–5, 6–10, and 11–14 were considered poor, fair, and good, respectively (30). The studies were evaluated by two experts independently; any conflict of opinion was discussed or consulted by a third expert and resolved.

## 3 Results

### 3.1 Overview of the included studies

#### 3.1.1 Study search

**Figure 1:**
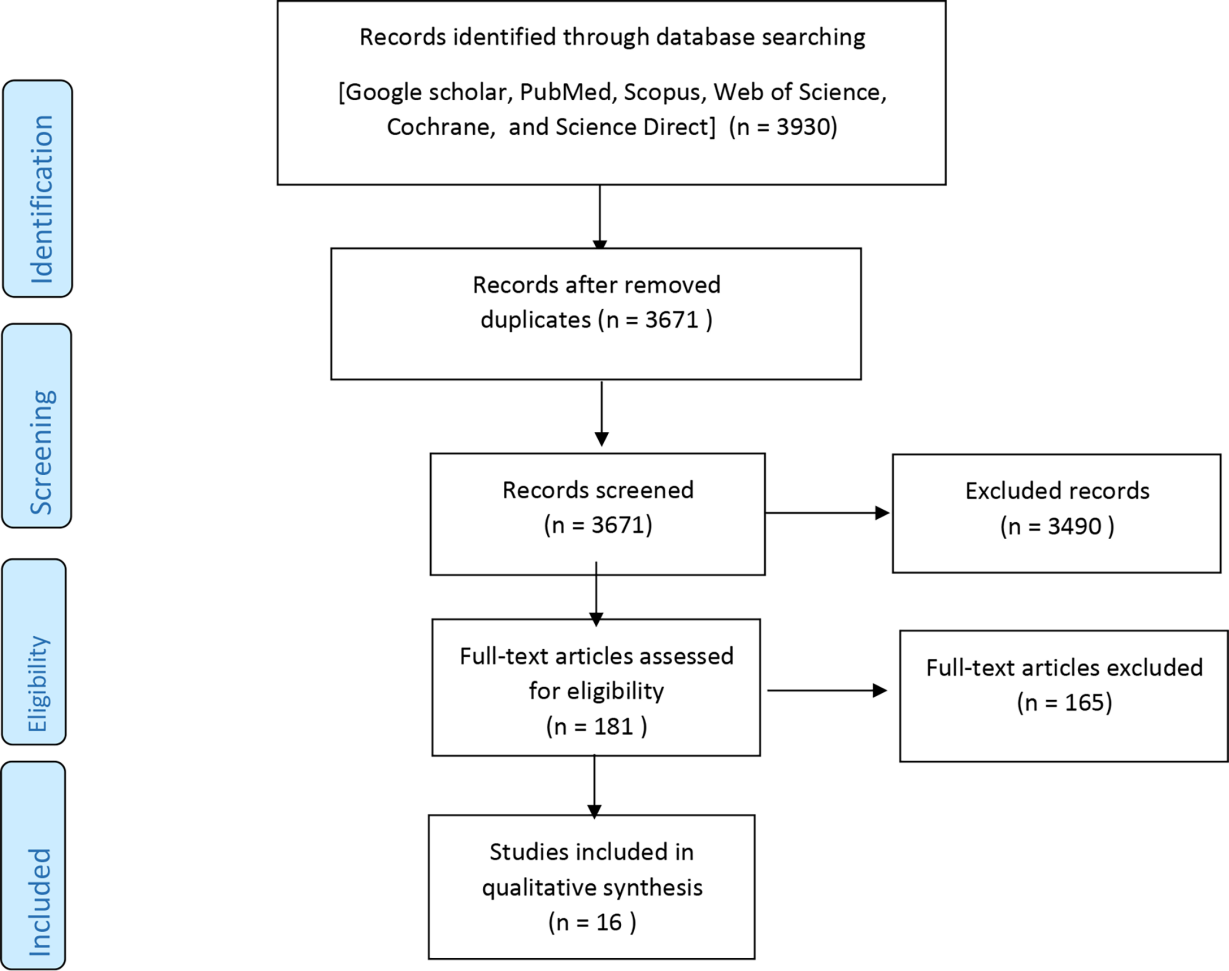
Search method and study selection.

Database search resulted in 3932 records, of which 3490 were primarily excluded based on title and abstract. The remaining 181 articles were thoroughly studied and the articles that met the inclusion criteria were extracted, leaving us with 16 studies. The remaining articles were carefully evaluated for qualitative assay. **Figure 1** presents the steps of the study selection in more detail.

#### 3.1.2 Study characteristics

Included studies dated from 2021 to 2022. Sample sizes of the studies varied from 86 to 56261. The mean age of the participants ranged from 29 to 70.38. The proportion of males ranged from 27.8% to 65.9% in the studies. The number of patients with DM was mentioned in 15 of the articles and ranged from two to 4626 (27–35, 37–42). One study’s population consisted of DM patients, but the number was not mentioned (27). Eleven of the studies had controls (27, 29, 30, 32–35, 37, 38, 41, 42), while five of them did not (27, 28, 31, 39, 40). Type of diabetes was specifically mentioned in six studies; four were T2DM (27, 32, 34, 42), and two were both T1DM and T2DM (35, 38). In terms of the type of vaccine; BNT162b2 was assessed in twelve of the studies (27, 28, 30, 32, 33, 35, 37–39, 41, 42), mRNA-1273 was assessed in two of the studies (32, 35), CoronaVac, Sinopharm or SinoVac were assessed in four studies (28, 29, 31, 39), ChAdOx1-S was assessed in four studies (32, 34, 35, 40). Ad5-nCoV (30) and BBV-152 (34) were also assessed in a few studies.

**Table 1** presents the detailed characteristics of the included studies.

**Table 1:** Characteristics of the included studies.

Study (Author, year)	Country	Study design	Number of patients with DM	Number of controls	Mean age	Proportion of male (%)	Type of DM	Type of vaccine	Anti-bodies outcome	Follow-up period	Dose	Number of days between doses	Cut-off for positive seroconversion
Ali,H. et al, 2021 (43)	Kuwait	cohort	81	181	49.3	51.9	T2DM	BNT162b2	IgG IgM Neutralizing antibodies	minimum of 3 weeks	2	NA	> 31.5 binding antibody units (BAU)/ml
Alqassieh, R. et al, 2021 (28)	Jordan	prospective observational cohort	76	NA	NA	65.6	NA	BNT162b2 Sinopharm	IgG IgM	6 weeks	2	21 days	index ≥ 1 (index is defined as a ratio between the relative fluorescence value (RFV) measured in the sample and the RFV obtained for the calibrator)
Güzel et al, 2021 (29)	Turkey	Prospective cohort	80	103	37.2	46.4	NA	CoronaVac-SinoVac	IgG	21 days	2	28 days	AU>1.1 (the ratio between the optical density of the sample and the optical density of the negative control expressed as arbitrary units (AU).
Guzmán-Martínez,O. et al, 2021 (30)	Mexico	Cohort	14	101	55.9	34.8	NA	BNT162b2 Ad5-nCoV	Anti-S1 IgG	BNT162b2 : 3-4 weeks after the first dose 2-3 weeks after the second dose Ad5-nCoV: 5-6 weeks	BNT162b2: 2 Ad5-nCoV: 1	NA	index ≥ 1.1 (index is calculated by dividing the value of the optical density (OD) of each serum by the value of the OD calibrator.)
Lustig et al, 2021 (37)	Israel	longitudinal cohort study	139	2496	47.7	27.8	NA	BNT162b2	IgG IgA Neutralizing antibodies	1-2 weeks after the first and second vaccine dose	2	3 weeks	IgG > 0·62 sample-to-cutoff (s/co) ratio. IgA>1·1 s/co. level of neutralizing antibodies >10 were.
Nomura,Y. et al, 2021 (33)	Japan	single-centre prospective observational study	12	353	44	32.5	NA	BNT162b2	Anti-spike antibody IgG	3 months	2	3 weeks	Not reported
Saure et al, 2021 (39)	Chile	surveillance study	4626	NA	NA	41.1	NA	CoronaVac BNT162b2	IgG IgM	16 weeks after the second dose	2	NA	visible bands on the IgG and test control positions
Singh A. K. et al, 2021 (34)	India	Cross-sectional	57	495	44.85	59.2	T2DM	ChAdOx1-nCOV BBV-152	Anti-spike antibody IgG	6 months	2	NA	> 15.0 AU/mL
Van Praet et al,2021 (41)	Belgian	case control	25	75	41.5	53	NA	BNT162b2					50 AU/mL
Watanabe et al, 2021 (42)	Italy	observational study	2	66	29	39.5	T2DM	BNT162b2	anti-S1-RBD IgGs	1–4 weeks after the second inoculation.	2	3 weeks	Not reported
Karamese, M. et al, 2022 (31)	Turkey	cross sectional study	49	NA	70.38	52.8	NA	CoronaVac	Anti-SARS-CoV2-antibodies	4 weeks	2	NA	>35.2 IU/ml (a ratio of the optical density (OD) of the samples over the OD of the calibrators)
Marfella, R. et al, 2022 (32)	Italy	prospective observational study	201	277	57.3	55.6	T2DM	BNT162b2 ChAdOx1-S mRNA-1273	IgG	4 weeks	2	NA	≥20% inhibited binding of the anti-IgG-horseradish peroxidase (HRP)-RBD to ACE2 receptors, compared to control.
Papadokostaki, E. et al, 2022 (38)	Greece	prospective observational study	58	116	51.3(control) 52.6(DM)	38.5	T1DM, T2DM	BNT162b2	Neutralizing antibodies	21 days 52 days	2	BNT162b2: 21days ChAdOx1-S: 28-52 days mRNA-1273 :28 days	>50 AU/mL
Sourij, C. et al, 2022 (35)	Austria Germany	prospective, multicenter cohort	161	86	49.2	54.7	T1DM, T2DM	BNT162b2 ChAdOx1-S mRNA-1273	Anti-SARS-CoV-2 RBD-IgG	21 days after the first dose. 7–15 days after the second dose. 70–75 days after the second and before the third dose of the vaccine.	3	NA	>0.8 U/mL
Tawinprai et al, 2022 (40)	Thailand	prospective cohort study	11	NA	40	34.9	NA	ChAdOx1	anti-RBD antibody	7-14 days after the first and 14-21 days after the second vaccination	2	NA	>0.8 U/mL
Terpos, E. et al, 2022 (27)	Greece	prospective observational study	NA	NA	48	32.9	NA	BNT162b2	neutralizing antibodies anti-S-RBD IgGs	3 months	2	21	NA

NA, not available.

#### 3.1.3 Quality assessment of the studies

Quality assessment of the included studies is presented in **Table 2**. The majority of the studies (n =9) (28, 30, 32–35, 39, 40, 43) were of good quality and seven (27, 29, 31, 37, 38, 41, 42) had fair quality.

**Table 2:** Quality assessment of the included studies.

Study	Total score	Q1	Q2	Q3	Q4	Q5	Q6	Q7	Q8	Q9	Q10	Q11	Q12	Q13	Q14
Ali,H. et al, 2021 (43)	11	Yes	Yes	Yes	Yes	No	Yes	Yes	NA	Yes	Yes	Yes	NR	Yes	Yes
Alqassieh, R. et al, 2021 (28)	13	Yes	Yes	Yes	Yes	Yes	Yes	Yes	Yes	Yes	Yes	Yes	NR	Yes	Yes
Güzel et al, 2021 (29)	10	Yes	Yes	Yes	Yes	No	Yes	Yes	NA	Yes	Yes	Yes	NR	Yes	No
Guzmán-Martínez,O. et al, 2021 (30)	11	Yes	Yes	Yes	Yes	No	Yes	Yes	Yes	Yes	Yes	Yes	NR	Yes	No
Lustig et al, 2021 (37)	10	Yes	Yes	Yes	Yes	No	Yes	Yes	NA	Yes	Yes	Yes	NR	Yes	No
Nomura,Y. et al, 2021 (33)	11	Yes	Yes	Yes	Yes	No	Yes	Yes	NA	Yes	Yes	Yes	NR	Yes	Yes
Saure et al, 2021 (39)	12	Yes	Yes	Yes	Yes	No	Yes	Yes	Yes	Yes	Yes	Yes	NR	Yes	Yes
Singh A. K. et al, 2021 (34)	11	Yes	Yes	Yes	Yes	No	NA	Yes	Yes	Yes	Yes	Yes	NR	Yes	Yes
Van Praet et al,2021 (41)	10	Yes	Yes	Yes	Yes	No	Yes	Yes	NA	Yes	Yes	Yes	NR	Yes	No
Watanabe et al, 2021 (42)	10	Yes	Yes	Yes	Yes	No	Yes	Yes	NA	Yes	Yes	Yes	NR	Yes	No
Karamese, M. et al, 2022 (31)	9	Yes	Yes	Yes	Yes	No	Yes	Yes	NA	Yes	Yes	Yes	NR	No	No
Marfella, R. et al, 2022 (32)	12	Yes	Yes	Yes	Yes	Yes	Yes	Yes	Yes	Yes	Yes	Yes	NR	Yes	No
Papadokostaki, E. et al, 2022 (38)	10	Yes	Yes	Yes	Yes	No	Yes	Yes	NA	Yes	Yes	Yes	NR	Yes	No
Sourij, C. et al, 2022 (35)	12	Yes	Yes	Yes	Yes	No	Yes	Yes	Yes	Yes	Yes	Yes	NR	Yes	Yes
Tawinprai et al, 2022 (40)	11	Yes	Yes	Yes	Yes	No	Yes	Yes	NA	Yes	Yes	Yes	NR	Yes	Yes
Terpos, E. et al, 2022 (27)	10	Yes	Yes	Yes	Yes	No	Yes	Yes	NA	Yes	Yes	Yes	NR	Yes	No

NR, Not reported

### 3.2 Immunogenicity properties

#### 3.2.1 Seroconversion after COVID-19 vaccination in patients with DM

Several studies have been conducted on the immunogenicity of mRNA vaccines in patients with DM, most of which were indicative of significantly lower antibody response in patients with DM compared to those without (27, 32, 33, 35, 37–39, 41, 43). However, few studies showed no statistical difference (28, 30, 42). Ali et al. (43) evaluated anti-SARS-CoV-2 IgG and neutralizing antibodies after two doses of BNT162b2 mRNA vaccine in people with and without diabetes. Their results have indicated that although both groups of participants had high seropositivity three weeks after the second dose, the mean levels of IgG (154 ± 49.1 *vs.* 138 ± 59.4 BAU/ml) and neutralizing antibodies (87.1 ± 11.6 *vs.* 79.7 ± 19.5%) were significantly lower in patients with T2DM. Another study by Nomura et al. (33) has discovered similar immunogenicity of mRNA vaccines among diabetics compared to controls. The results revealed a significant association between DM and lower Ab titers against SARS-CoV-2 Spike Antigen. Median Ab titers (IQR) were 382 (211–741) and 768 (436–1150) in diabetics and non-diabetics, respectively (p-Value = 0.0189). However, this association was no longer significant after adjustment for age. In a recent work by Terpos et al. (27), it is found that BNT162b2 vaccine effectiveness declined significantly for people with diabetes compared to those with other comorbidities such as cardiovascular and autoimmune diseases (p-Value = 0.039). A prospective, single-center, longitudinal cohort study in Israel, has demonstrated that diabetes is significantly associated with lower concentrations of IgG antibodies (OR: 0.92; 95% CI: 0·39–2·19; p = 0·855) and non-reactive response of IgA antibodies (OR: 0.30; 95% CI: 0.13–0.73; p = 0.008) after BNT162b2 COVID-19 vaccination (37). In addition, Papadokostaki et al. compared the immunogenicity of SARS-CoV-2 BNT162b2 vaccine in in Greece patients with and without DM (38). Their analysis showed that about 17% of patients with DM had an insufficient immune response to the BNT162b2 mRNA vaccine after the first dose.

In contrast, their seroconversion rate after the second dose was adequate, and similar to those without DM and it remained high even after two months following the second dose. Moreover, Van Praet and his colleagues in Belgium have reported a reduced cellular response to BNT162b2 vaccine in patient with DM (p= 0.008), whereas the humoral response was not significantly associated with DM(p=0.135) (41). In contrast to the aforementioned studies, Alqassieh et al. (28) have found no statistical significance in Pfizer-BioNTech vaccine immunogenicity among patients with DM compared to control. Another recent study in Mexico compared the effectiveness and safety of BNT162b2 mRNA vaccines and adenovirus vector Ad5-nCoV vaccine (30). The results demonstrated no statistical significance between S1 IgG antibody titers and DM. Similar results have been reported in another single‐center observational study in Italy (42). According to this study, DM was not significantly associated with anti-SARS- CoV-2 antibody titer following BNT162b2 vaccination (p=0.876).

Several studies have been conducted on vector based vaccines, most of which showed significantly lower antibody response among patients with DM compared to those without (32, 34, 35, 40) and one of the studies revealed no statistical significance (30). The analysis of a cross-sectional study in India after adjustment for age, sex and BMI, showed that patients with T2DM had lower SARS-CoV-2 anti-spike antibody seropositivity rate in comparison to those without (79.6%, 37.5% after first dose of CovishieldTM and CovaxinTM in patients with T2DM in comparison to 87.7% and 44.3% in those without DM, respectively). A second dose of CovishieldTM and CovaxinTM resulted in 91.3% and 33.3% of individuals with DM being seropositive, respectively, compared to 98.9% and 83.3% of individuals without DM (34). Further, a prospective cohort study in Thailand among 796 participants (11 with DM) revealed that diabetes might affect the immunogenicity of ChAdOx1 COVID-19 vaccine as well as age, sex, and hematologic disease (40). Patients with diabetes had 55% (95% CI; 23%-84) lower anti-RBD antibody levels. Adjusted linear regression analysis with age, sex, and underlying comorbidity, showed that diabetes might be an influencing factor of anti-RBD antibody concentration (p=0.45, 95%CI =0.26–0.77). Contrary to these findings, Guzmán et al. found no significant relation between DM and seroconversion rate after COVID-19 vaccination (30). The level of S1 IgG antibody measured five to six weeks’ post-vaccination, was not significantly different between patients with DM compared to those without.

Inactivated vaccines have lower immunogenicity in DM patients than those without (29, 34, 39). One of the studies did not show any significant difference (28). Güzel et al. (29) designed a prospective study to investigate the seroconversion rate of CoronaVac-SinoVac vaccine. The results illustrated that antibody titers differed significantly in individuals with DM compared to those without, 3 weeks after the second dose (p<0.001). In another study conducted in Turkey, the participants with diabetes had significantly lower antibody responses compared to those without (p<0.001) (31). In addition to this, Singh et al. (34) has assessed the ChAdOx1-nCOV and BBV-152(Covaxin) vaccines’ immunogenicity in India. They showed that patients with T2DM had significantly lower antibody responses even after adjustment for confounders. Sauré et al. also conducted a surveillance study which shows lower IgG seroconversion rate in CoronaVac vaccine recipients than BNT162b2 vaccine recipients 1-4 weeks after the first dose and 5-9 weeks following the second dose (p<0·0001) (39). In contrast to the previous studies, Alqassieh et al. (28) have conducted a study in Jordan showing no statistically significant difference in IgG antibody levels in the collected blood samples of patients with DM compared to those without.

The immunogenicity outcomes of the included studies are detailed in **Table 3**.

**Table 3:** Outcomes of the included studies in DM patients *vs.* patients without DM.

Study (Author, year)	Seroconversion in patients with DM	Seroconversion in controls	Study findings
Ali,H. et al, 2021 (43)	Mean IgG: 138 (59.4) IgM: 58.1 (112) Neutralizing antibodies %: 79.7 (19.5)	Mean IgG: 154 (49.1) IgM: 65.6 (84.2) Neutralizing antibodies%: 87.1 (11.6)	Both SARS-CoV-2 IgG and neutralizing antibodies titers were significantly lower in people with T2DM compared to those without. Whereas no statistical significance was found between seroconversion and age, gender, obesity, and hypertension.
Alqassieh, R. et al, 2021 (28)	Percentage of positive seroconversion IgG: 84.2%	NA	More than 50% of participants with negative seroconversion were diabetics. Despite of the diminishing impact of diabetes mellitus on IgG levels, this effect was not statically significant.
Güzel et al, 2021 (29)	NA	NA	people with DM had significantly lower antibody response levels compared with those without DM
Guzmán-Martínez, O. et al, 2021 (30)	Mean S1 IgG indices BNT162b2: 6.93 Ad5-nCoV: 2.86	Mean S1 IgG indices BNT162b2: 8.25 Ad5-nCoV: 4.26	No statistical significance was found between generation of S1 IgG antibodies and diabetes.
Lustig et al, 2021 (37)	NA	NA	IgG and detectable IgA antibody levels were lower in patients with DM (OR: 0.92; 95% CI: 0·39–2·19; p = 0·855) And OR: 0.30; 95% CI: 0.13–0.73; p = 0.008 respectively). The rate of positive neutralization was not significantly lower in individuals with DM compared to those without (OR: 0.53; 95% CI: 0·21–1·30; p = 0·166). Endpoint regression analysis demonstrate lower antibody response in diabetes group. (ratios of means of IgG and neutralizing antibody titers 3 weeks after the first dose:1·03 (0·80–1·32) and 0·83 (0·50–1·38), respectively. And at 3-5 weeks for IgG and 3-4 weeks for neutralizing antibodies: 0·84 (0·62–1·14) and 0·70 (0·38–1·28), respectively.)
Nomura,Y. et al, 2021 (33)	Antibody titer, Median: 382	Antibody titer, Median: 767	Diabetes mellitus was significantly associated with a lower antibody titer.
Saure et al, 2021 (39)	Seropositivity: After 1st dose of Sinovac: 17.3% pfizer: 40.5% 4 weeks after 2nd dose sinovac:58.0% pfizer: 89.3 8 weeks after 2nd dose sinovac:60% pfizer: 92.5%	NA	Diabetes was related to low seropositivity among CoronaVac recipients.
Singh A. K. et al, 2021 (34)	Percentage of positive seroconversion; First dose: 73.7%	Percentage of positive seroconversion; First dose : 80%	People with T2DM had a significantly lower seropositivity rate compared to those without.
		Percentage of positive seroconversion; Second dose : 96.1	
Van Praet et al,2021 (41)	NA	NA	A significant association between diabetes and reduced cellular response has been found (Estimate 95% CI= −0.297 (−0.515 to −0.079) and P Value= 0.008). However, diabetes had no significantly effect on humoral response (P Value= 0.135)
Watanabe et al, 2021 (42)	NA	NA	No statistical significance was found between anti-SARS- CoV-2 antibody titer and diabetes. (p=0.876)
Karamese, M. et al, 2022 (31)	NA	NA	The participants with diabetes had significantly lower antibody responses vs those without.
Marfella, R. et al, 2022 (32)	NA	NA	Neutralizing antibodies and antigen-specific CD4+ T-cell responses were reported in this study, 21 and 52 days after the second vaccine dose.
Papadokostaki, E. et al, 2022 (38)	Anti-SARS-CoV-2 RBD IgG titers in AU/mL Geometric mean (95% confidence intervals); At 21 days after the first dose: 220.10 (122.59, 395.17) At 7–15 days after the second dose: 5300.64 (3868.71, 7262.56) At 70–75 days after the second: 1246.77 (853.76, 1859.89)	Anti-SARS-CoV-2 RBD IgG titers in AU/mL Geometric mean (95% confidence intervals); At 21 days after the first dose: 354.62 (268.34, 468.65) At 7–15 days after the second dose: 6281.32 (5244.47, 7523.16) At 70–75 days after the second: 1677.94 (1412.94, 1991.53)	Seventeen percent of participants with DM had inadequate humoral immune response after the first dose; however, after the second dose both participants with and without DM developed high and similar seroconversion.
Sourij C. et al, 2022 (35)	Percentage of positive seroconversion; First dose: T1DM: 52.7 T2DM: 48 Percentage of positive seroconversion after second dose was similar to first dose.	NA	Higher antibodies levels had been reported among T1DM patients in comparison to non-diabetics and T2DM patients, 14 to 21 days after the second dose.
Tawinprai et al, 2022 (40)	NA	NA	Participants with diabetes had 55% (95%CI; 23%-84%) lower anti-RBD antibodies level.
Terpos, E. et al, 2022 (27)	NA	NA	At the day after the second dose, comorbidities like diabetes, cardiovascular problems, and autoimmune diseases had negative impact on neutralizing antibody levels; however, the effect of diabetes on low seroconversion was statistically more than others.

NA, not available.

#### 3.2.2 Immunogenicity of COVID-19 vaccination in patients with DM based on DM characteristics

Several studies have assessed the correlation between additional variables (i.e., age, gender, type of DM, glycemic control, BMI, eGFR, comorbidities such as hypertension, type of diabetes management, insulin therapy) and antibody response in patients with DM *vs.* patient without DM (32, 34, 35, 38, 43).

A study by Marfella et al. (32) with a prospective observational design was conducted to investigate the association between glycemic control and vaccine immunogenicity. Neutralizing antibodies and antigen-specific CD4-cell responses were assessed 21 and 52 days after the second vaccination dose. Interestingly, the results from this study have shown a direct association between HbA1c levels and the immunological responses to ChAdOx1-S vaccines. DM patients with inadequate glycemic control (HbA1c>7%) had lower neutralizing antibodies and antigen-specific CD4+ T-cell responses compared to non-diabetics and diabetics with sufficient glycemic control (HbA1c>7%). This is while three other studies have shown no significant association between glycemic control and antibody response in DM (34, 35, 38). Of note, Sourij et al. showed that Anti-SARS-CoV-2 S levels after ChAdOx1-S vaccine was not significantly different between poorly controlled and well controlled T1DM patients (P=0.249). This was also true among patients with T2DM. Moreover, according to their results, baseline HbA1c levels or its changes during the follow up were not significantly correlated with antibody response among DM patients (r=-0.07, p=0.398). Sourij et al. also assessed the association between type of DM and antibody response showing higher seroconversion among T1DM patients compared to T2DM patients (P=0.013). 52.7% of T1DM patients and 48.0% of T2DM patients were seropositive 1 to 2 weeks after the first vaccination. Well controlled T1DM had a higher seropositivity rate in comparison to patients with well or poorly controlled T2DM patients,14-21 days after the second vaccination (p=0.003 and p=0.034, respectively). However, this difference did not remain significant when adjusted for confounders (35).

Three studies have assessed the association between age, gender, and BMI with antibody response in DM patients (35, 38, 43). There was no significant association between either age, gender, or BMI and antibody response in the study by Ali et al. (43). In two other studies, the antibody response decreased with age. The results from the study by Papadokostaki et al. showed that age was significantly correlated with RBD-IgG levels (r = -0.327, p = 0.020) 21 days after the first dose, however this association was no longer significant 7-15 days and 70-75 days after the second dose of the vaccine (38). Age and BMI were also negatively correlated with Anti-SARS-CoV-2 S antibody response in the study by Sourij et al. (r=- 0.45, p<0.001 and r = -0.18, P = 0.027, respectively). The association between age and antibody response was stronger among T1DM compared to T2DM (35).

Type of diabetes management and insulin therapy and how they affect the antibody response in patients with DM were assessed in two studies (34, 35). In the study by Singh et al. (34), diabetes management was divided into monotherapy, combination therapy, insulin therapy, and no medication; there was no significant association between type of diabetes management and antibody response in DM patients of this study (34). Similarly, an earlier study found no significant association between insulin therapy and antibody response among patients with diabetes (35).

Duration of diabetes and its association with antibody response was assessed among patients with DM in three studies, none of which showed any significant association (34, 35, 38). Moreover, interestingly the study by Sourij et al. revealed a significant positive correlation between estimated glomerular filtration rate (eGFR) and antibody response among patients with DM (r=0.28, P=0.001) (35).This finding also approved in other studies (44–46). In the Ali et al. study, hypertension and previous COVID infections were also assessed, but no significant correlation was found (43). Outcomes of the included studies based on DM characteristics are provided in **Table 4** in more detail.

**Table 4:** Outcomes of the included studies based on diabetic characteristics.

Study (Author, year)	Assessed variables	Association between the variables and immunogenicity
Ali,H. et al, 2021 (43)	Age	Age (above or below 60) had no significant interaction with the effect of DM on antibody response (P=0.103).
	Gender	Gender did not show any significant interaction with the effect of DM on antibody response (P=0.634).
	BMI	BMI (above or below 30) had no significant effect on the association between DM and antibody response (P=0.563)
	Hypertension	Hypertension had no significant interaction with the effect of DM status on antibody response (P=0.393).
	Previous COVID infection	Previous COVID infection, did not significantly affect the antibody response among patients with DM compared to patient without DM.
Singh A. K. et al, 2021 (34)	Duration of diabetes	Percentage of positive seroconversion was 81.8% and 100% among patients with DM duration < 5 years, 67.4% and 76.7% among those with 5–10 years’ duration and 73.7% and 92.9% among patients with over 10 years of DM, after first and second dose respectively. There was no statistically significant difference between the subgroups.
	Glycemic control	Percentage of seroconversion was 72.7% and 84.3% in DM patients with optimum glycemic control and 0% and 100% in those with poor control, after first and second dose respectively. There was no statistically significant difference between the subgroups.
	Diabetes management	Percentage of seroconversion was 87.5% in DM patients with monotherapy, 68.6% and 93.3% and 81.2% in those with combination therapy, 100% among patients on insulin and 60% and 80% among DM patients with no medication, after first and second dose respectively. There was no statistically significant difference between the subgroups.
Marfella, R. et al, 2022 (32)	Glycemic control and HbA1c	Patients with poor glycemic control (HbA1c >7%) has significantly lower levels of neutralizing antibody levels compared to patients with good glycemic control (HbA1c <7%).
Papadokostaki, E. et al, 2022 (38)	Age	Twenty-one days after the first dose, age was significantly correlated with RBD-IgG levels (r = -0.327, p = 0.020), however this association was no longer significant 7-15 days and 70-75 days after the second dose of the vaccine.
	Duration of diabetes	There were no significant correlation between duration of diabetes antibody response in patients with DM after the first or the second dose of the vaccine.
	HbA1c	HbA1c had no significant correlation with antibody response in patients with DM status after the first or the second dose of the vaccine.
Sourij C. et al, 2022 (35)	Age	Age had a moderate to strong negative correlation with antibody response (r= -0.45, P < 0.001) in patients with DM. This association was significant and stronger among patients with T1DM compared to patients with T2DM (r= -0.53, P < 0.001 *vs.* r= -0.20, P=0.087).
	Gender	Gender was not significantly associated with antibody response in patients with DM.
	BMI	BMI of patients with DM had a weak negative correlation with antibody response (r = -0.18, P = 0.027).
	Type of DM	T1DM patients had higher seroconversion compared T2DM patients (P=0.013), however this association did not remain significant after adjusting for age and sex.
	Glycemic control and HbA1c	Seroconversion was not significantly associated with glycemic control (HbA1c cut off point=58mmol/l) among either T1DM or T2DM patients. Antibody response in patients with DM was not significantly correlated with either baseline HbA1c levels (r = 0.07, P = 0.398) or changes of HbA1c levels between baseline and the follow-up visit after the second dose of the vaccine (r = 0.06, P = 0.509).
	Insulin therapy	Insulin therapy was not significantly associated with seroconversion in patients with T2DM.
	Duration of diabetes	Duration of diabetes was not significantly associated with antibody response in patients with DM.
	GFR	There was a significant positive association between eGFR and antibody response among patients with DM (r=0.28, P=0.001).

## 4 Discussion

Since the emergence of COVID-19 pandemic and the development of vaccines against the virus, immunogenicity has gained increasing attention as an indicator of vaccine effectiveness (47). Immunogenicity is depicted by observing the binding and neutralizing antibodies produced after a total dose of vaccine (48). Upon vaccination, mRNA and adenovirus vector vaccines encoding the SARS-CoV-2 spike (S) protein enter dendritic cells, leading to production of high levels of S protein, and inactivated vaccines contains the whole non-infective virus particles and adjuvants which are directly ingested and processed by antigen presenting cells. Besides that, intrinsic adjuvants inside the vaccine activate innate immune system by producing type I interferon and multiple pro-inflammatory cytokines and chemokines. Hence, antigen and co-stimulatory molecules are introduced by the activated dendritic cells to S protein-specific naive T cells, which become activated and form the effector cells to generate cytotoxic T lymphocytes or helper T cells. The S protein-specific B cells differentiate into antibody-secreting plasma cells with the assistance of T helper cells, which results in production of high affinity anti-S protein antibodies. After vaccination, these T cells and B cells counteract infection with SARS-CoV-2. Most of the cases develop neutralizing antibodies rapidly after infection with SARS-CoV-2. Antibodies against the spike receptor-binding domain (RBD) or the amino terminal domain (NTD) of the spike protein have effective SARS-CoV-2-neutralizing activity (49–51). Furthermore, COVID-19 vaccines induce T cell responses which can be evaluated by IFNγ release, IL-2 release, or both (52, 53).

Several studies illustrated that the immune process might be inadequate in people with diabetes (54). Numerous factors may play a role in increasing the susceptibility of DM patients to the severity and complications of COVID-19. Diabetes as a metabolic disorder generates a chronic, systemic low-grade inflammation. Consequently, after COVID-19 antigen exposure, this metabolic inflammation may impair macrophage activation, exaggerate pro- inflammatory cytokines/chemokines like TNF-a, IFN-g and alter innate/adaptive immunity (55–58). Moreover, B and T cell responses are altered in people with diabetes in several ways for instance, reduced expression of co-stimulatory molecules (CD69, CD28, CD40 ligand) or interleukin-12 receptor on T cells which leads to lower production of interferon and granzyme B (59, 60). Therefore, all these mechanisms hamper the immune system and make people with diabetes at higher risk of adverse COVID-19 outcomes. As a result, this population is prioritized to receive COVID-19 vaccinations. COVID-19 vaccination is necessarily considered in this population. Despite the low immunogenicity of hepatitis B vaccine and inconclusive results to other vaccines, such as influenza, varicella zoster, and pneumococcus, more research is needed to determine SARS-CoV-2 vaccine immunogenicity in this group (61–63).

In this systematic review, most of the studies showed a significantly lower antibody response in patients with DM than those without. In one prospective observational study, 17% of participants with DM had an inadequate humoral immune response to SARS-CoV-2 BNT162b2 Vaccine after the first dose; however, after the second dose, both participants with and without DM developed high and similar seroconversion (38). One study discovered significantly lower levels of antibodies after full BNT162b2 vaccination among people with diabetes. While this significance does not remain after the age adjustment of analysis (33).

In another study, Sourij et al. have observed high immune response in patients with T1DM compared to patients with T2DM and healthy controls, according to the unadjusted analyses. They also extracted relevant variables, including the patients’ mean age, sex, BMI, estimated glomerular filtration rate (eGFR), and other comorbidities. It has been found that among patients with type 1 or 2 diabetes, age and eGFR directly affected anti-SARS-CoV-2 S antibody response, whereas baseline HbA1c levels or its changes during the follow up did not. In addition, BMI of patients with DM had a weak negative correlation with antibody response (35). Although, in this study, the T1DM group had higher antibody levels than T2DM, this result did not remain significant after adjustment for age and sex. Regarding the results of this study, age and obesity had an inverse impact on vaccine immunogenicity (35). This notion is supported by previous studies demonstrating lower seroconversion of vaccines in elderly people and the inverse influence of BMI on vaccine response (64, 65). A possible explanation for the heterogeneity of the results of included studies might be that the average age and BMI were higher in the T2DM group than in T1DM. Another explanation could be lower mean eGFR in the T2DM group; impaired renal function might negatively affect the vaccine immunogenicity in T2DM patients compared to T1DM. This hypothesis agrees with previous data showing low seropositivity after vaccination in patients with chronic kidney disease (35, 66–69). Moreover, higher rate of other comorbidities such as hypertension, coronary heart disease, myocardial infarction among T2DM group might confound the results (42, 70–72).

Nevertheless, the majority of studies in this systematic review confirm the effect of diabetes on vaccine immunogenicity. In one study, no significant difference was found in antibody titers between people with diabetes and those without. Guzmán and his collogues also reported no statistically significant association between S1 IgG antibodies generation and diabetes (30). This report is in line with two other studies which show no statistical difference in serological response following vaccination among people with diabetes (28, 42). In order to explain these discrepant results, several points should be considered. Firstly, these three studies, which have reported no significant results, were carried out through a general population, not specifically the diabetic population.

Furthermore, the number of diabetic patients included in these studies was low. For instance, Watanabe et al. include only two participants with diabetes (42). Hence the results cannot be extrapolated to all the patients with DM. Secondly, the clinical characteristics of the diabetic subgroup in these studies were insufficient to explain the heterogeneity of the immune response. The confounding factors such as mean age and BMI of people with diabetes, type of diabetes, duration, and management have not been mentioned in these studies, possibly hindering some results. Thirdly, they have assessed only the humoral component of the immune system, specifically IgM or IgG, and have not measured neutralizing antibodies and the T- cell immune response; This might partly explain the incoherent results of these studies compared to others. Fourthly, it is noteworthy that the time of collection and evaluation of samples ranged between 1 and 6 weeks following the second vaccination dose, introducing time as a possible bias. The follow-up duration in some other studies in our systematic review reached six months. As a result, they could provide more detailed information about antibody or immunity decay. Finally, considerable variability exists among available SARS-CoV-2 antibody tests, which might lack adequate sensitivity to estimate antibody response and consequently leads to discrepant results in these three studies compared to other studies.

Nevertheless, it remains unclear whether the reduced response to the vaccine in diabetics results from a quantitatively lower immune reaction or is associated with poor clinical efficacy. Studies have shown an inherently interrelated relationship between immunity and metabolism. Therefore, diabetes, as an ingredient of metabolic disorders, induces immune defects (73). It appears that people with diabetes show a lower immune response after COVID vaccination. Impairment in lymphocyte proliferation, dysregulation of monocyte/macrophage and neutrophil function, reduced antigen presentation, and deteriorated complement function, followed by hyperglycemia and insulin resistance, could justify lower levels of antibodies in diabetes (74, 75). Marfella et al. also examine the effects of glycemic control on the immunogenicity of m-RNA and vector-based vaccines in patients with type 2 diabetes. The results showed that hyperglycemia impairs adaptive immunity and virus-neutralizing antibodies, resulting in insufficient vaccine immunity against Covid-19. Both neutralizing antibodies and antigen-specific CD4+ T-cell responses were significantly lower in T2DM patients with insufficient glycemic control (HbA1c>7%) compared to the others (32). However, whether hyperglycemia alters the immune responses of vaccines is still a matter of debate. A study of 150 patients in Italy consisting of 26.6% diabetics showed no significant negative effect of hyperglycemia on immune responses. They have reported that neutralizing antibody response to SARS-CoV-2 in participants with diabetes is similar to those without (25, 26). Contrarily, many studies have discovered an inverse association between high glycemic levels and the immunogenicity of vaccines. Yelin et al. (76) have claimed that people with diabetes have lower seroconversion after administration of BNT162b2 (Pfizer) in comparison to non-diabetics; thus, precise glycemic control is recommended to achieve highly sufficient vaccine immunogenicity. Besides hyperglycemia, in a study reporting m-RNA and vector-based vaccine immunogenicity, other clinical features and comorbidities relevant to diabetes could play a critical role in vaccine immunogenicity, including age, estimated glomerular filtration rate, and body mass index (35).

Of note, Alqassieh et al. compared the effectiveness of two SARS- CoV-2 vaccines that are widely available, Sinpharm (the inactivated CoronaVac vaccine) and PfizereBioNTech’s (the mRNA BNT162b2 vaccine), among the adult population in Jordan. This study showed that after six weeks of the second dose, there was lower seropositivity among DM patients given the Sinpharm vaccine than Pfizer- BioNTech’s vaccine (28). In another study by Saure et al. lower seroconversion rate has been reported among DM patients given the Sinovac vaccine compared to those who received the Pfizer- BioNTech’s vaccine (39). It is worth noting that among the participants with comorbidities, such as chronic cardiovascular disease, chronic pulmonary disease, obesity, and cancer assessed in this study, the DM group had the lowest seropositivity response. Moreover, a study by Singh et al. also revealed that patients with T2DM had a significantly lower immunogenicity response than those without, both in Covishield and Covaxin recipients (34). In addition to this, another study determining the effects of glycemic control on immunological vaccine responses, administration of different types of vaccines made no remarkable changes in results, and diabetic patients who received mRNA vaccines (mRNA-BNT162b2 and mRNA-1273 vaccine) and viral vector-based vaccine (ChAdOx1-S), both had lower seroconversion (32).

Altogether, most studies show their acceptable efficacy in immunizing patients with DM against COVID-19. Although the heterogeneity of the outcome measures (some presented as mean and some others as percentages) has made the comparison difficult, but all together when pooling the conclusions from these thirteen studies, it could be inferred that despite the lower seroconversion in DM patients, there was still a considerable amount of antibody response to vaccines in patients with DM. However, due to its lower immunogenicity in DM compared to healthy subjects, a third or even fourth dose may be considered necessary for patients with DM to reach the same level of immunity as those without diabetes. This still remains a controversy and further research is warranted.

### Strengths and limitations

This study has some strengths. To the best of our knowledge, this is the first study to systematically review the immunogenicity of COVID-19 vaccines in patients with DM. Our data could help investigate the necessity of booster doses in this population to build successful vaccination strategies in the future. Additionally, this study provides important data about how glycemic control affects vaccine immunogenicity and other factors that could be considered before administering vaccines or during vaccination for people with diabetes.

This study has several limitations. Firstly, we could not perform meta-analysis due to the low number of included studies and the heterogeneity of the outcome measures. Secondly, we assessed the immunogenicity mostly based on the antibody response, which depends on the humoral immunity. While, cellular immunity is also involved in immunization after COVID-19 vaccination (77). Since the assessment of cellular immunity was not well established in the literature, the results from this review may not be fully reflect the protective effect of vaccination. Finally, the cut-off level of antibody indicating positive seroconversion is not univocal among included studies; this could have hampered possible significant results and may have affected the interpretation of our findings.

### Future direction

This systematic review revealed some literature gaps that could be addressed in the future. Firstly, there has been plenty of research on COVID-19 disease and vaccination in patients with DM, but there are limited studies on the immunogenicity of the vaccines in this group of high risk patients. We recommend further research focusing on seroconversion after COVID-19 vaccination in patients with DM. Secondly, most of the studies have used different types of vaccines without differentiating and comparing their immunogenicity, specifically in patients with DM. Further research on comparing different vaccine types (mRNA *vs.* vector-based *vs.* inactivated vaccines) is warranted. Thirdly, it is noteworthy that most of the studies have discussed all types of diabetes without differentiation. However, DM consists of different types with different pathophysiology and a wide range of symptoms and management strategies. Similar approach to different types of diabetes for making healthcare policies may not be cost-effective; thus, we recommend future research to assess and compare the immunogenicity of COVID-19 vaccines in different subgroups of DM. Lastly, it is noteworthy that based on our systematic literature search, there has not been any published original article evaluating the immunogenicity of the third dose of COVID-19 vaccines in patients with DM. Research in this regard is strongly recommended.

## Conclusion

In conclusion, vaccination reduces mortality and morbidity related to COVID-19, especially in high-risk groups like people with diabetes. Studies have elucidated that people with diabetes had lower antibody levels after two doses of vaccination, irrespective of the vaccine type. Furthermore, management of diabetes and glycemic control could be associated with antibody responses. On the contrary, some studies have found no significant difference in vaccine immunogenicity between patients with DM and the control groups. Hence, further studies are required to evaluate the immune response following COVID-19 vaccination in patients with diabetes and investigate whether booster shots are required to achieve a sufficient level of immunity in this specific population.

## Data availability statement

The original contributions presented in the study are included in the article. Further inquiries can be directed to the corresponding author.

## Author contributions

Conceptualization: AB and MF; methodology: FK and AS; validation: LG; data curation: PK; writing—original draft preparation: DN; writing—review and editing: all. All authors contributed to the article and approved the submitted version.

## Acknowledgments

Authors wish to thank all who helped in drafting this manuscript.

## Conflict of interest

The authors declare that the research was conducted in the absence of any commercial or financial relationships that could be construed as a potential conflict of interest.

## Publisher’s note

All claims expressed in this article are solely those of the authors and do not necessarily represent those of their affiliated organizations, or those of the publisher, the editors and the reviewers. Any product that may be evaluated in this article, or claim that may be made by its manufacturer, is not guaranteed or endorsed by the publisher.
